# Transcriptome profiling of pumpkin (*Cucurbita moschata* Duch.) leaves infected with powdery mildew

**DOI:** 10.1371/journal.pone.0190175

**Published:** 2018-01-10

**Authors:** Wei-Li Guo, Bi-Hua Chen, Xue-Jin Chen, Yan-Yan Guo, He-Lian Yang, Xin-Zheng Li, Guang-Yin Wang

**Affiliations:** School of Horticulture Landscape Architecture, Henan Institute of Science and Technology, Xin Xiang, China; Leibniz-Institut fur Naturstoff-Forschung und Infektionsbiologie eV Hans-Knoll-Institut, GERMANY

## Abstract

Cucurbit powdery mildew (PM) is one of the most severe fungal diseases, but the molecular mechanisms underlying PM resistance remain largely unknown, especially in pumpkin (*Cucurbita moschata* Duch.). The goal of this study was to identify gene expression differences in PM-treated plants (harvested at 24 h and 48 h after inoculation) and untreated (control) plants of inbred line “112–2” using RNA sequencing (RNA-Seq). The inbred line “112–2” has been purified over 8 consecutive generations of self-pollination and shows high resistance to PM. More than 7600 transcripts were examined in pumpkin leaves, and 3129 and 3080 differentially expressed genes (DEGs) were identified in inbred line “112–2” at 24 and 48 hours post inoculation (hpi), respectively. Based on the KEGG (Kyoto Encyclopedia of Genes and Genomes) pathway database and GO (Gene Ontology) database, a complex regulatory network for PM resistance that may involve hormone signal transduction pathways, transcription factors and defense responses was revealed at the transcription level. In addition, the expression profiles of 16 selected genes were analyzed using quantitative RT-PCR. Among these genes, the transcript levels of 6 DEGs, including bHLH87 (Basic Helix-loop-helix transcription factor), ERF014 (Ethylene response factor), WRKY21 (WRKY domain), HSF (heat stress transcription factor A), MLO3 (Mildew Locus O), and SGT1 (Suppressor of G-Two Allele of Skp1), in PM-resistant “112–2” were found to be significantly up- or down-regulated both before 9 hpi and at 24 hpi or 48 hpi; this behavior differed from that observed in the PM-susceptible material (cultivar “Jiujiangjiaoding”). The transcriptome data provide novel insights into the response of *Cucurbita moschata* to PM stress and are expected to be highly useful for dissecting PM defense mechanisms in this major vegetable and for improving pumpkin breeding with enhanced resistance to PM.

## Introduction

The genus Cucurbita is composed of several species, including the cultivated *C*. *moschata* (*Cucurbita moschata* Duch.), *C*. *pepo* (*Cucurbita pepo* L.), *C*. *maxima* (*Cucurbita maxima* Duch.) and several wild species. *Cucurbita moschata* is an economically important species that is cultivated worldwide. Pumpkins are valued for their fruit and seeds and are rich in nutrients such as vitamins, amino acids, flavonoids, phenolics and carbohydrates [1, 2]; pumpkins also have important medicinal properties, including antidiabetic, antioxidant, anticarcinogenic and anti-inflammatory activities [3, 4]. The mature fruit can be stored for 4 months or longer under proper conditions. Despite the economic importance of pumpkin, the latest available genomes (*C*. *maxima* and *C*. *moschata*) have only recently been made available [5]. This genome availability is unlike that of other cucurbits, such as watermelon (*Citrullus lanatus*), cucumber (*Cucumis sativus*) and melon (*Cucumis melo*), transcriptomes of which [6–8] whole-genome sequences have already been generated [9–11]. To date, research on pumpkin (*C*. *moschata*) (2n = 2x = 40), especially at the molecular level, remains at a low level, seriously hindering development in the fields of molecular biology and genetics.

Cucurbit powdery mildew (PM), mainly caused by *Podosphaera xanthii* (formerly *Sphaerotheca fuliginea*) [12, 13], is a serious biotrophic pathogen disease in field and greenhouse cucurbit crops worldwide. PM slows plant growth, and it causes premature desiccation of the leaves and a consequent reduction in the quality and marketability of the fruits. Breeding for PM resistance is the most desirable strategy to control this disease by means of resistant cultivars [14, 15]. Consequently, several genes involved in resistance to PM have been reported in different plant species [16, 17]. Thirteen *MLO*-like genes required for susceptibility to PM have been identified with the help of the published genome sequence of cucumber [18], and *CsaMLO8* has been characterized as a functional hypocotyl susceptibility gene to PM [19]. Gene expression differences in cucumber triggered by PM have been identified by comparative transcriptome profiling [20]. In addition, the expression of *CmMLO2* in muskmelon has been reported to be related to the pathogenesis of PM [21]. However, like non-model species, the molecular foundation of pumpkin is relatively weak. Transcriptome analysis of pumpkin during PM infection with a new generation of high-throughput sequencing technology is therefore necessary.

Next-generation sequencing (NGS) based RNA sequencing for transcriptome methods (RNA-Seq) have been proven to be an effective method to analyze functional gene variation, and NGS has dramatically improved the speed and efficiency of gene discovery. In recent years, RNA-Seq analysis has been applied to the transcriptomic profiles of species within the Cucurbitaceae family, such as *Citrullus lanatus* [6], *Cucumis sativus* [7], *Cucumis melo* [8], *Momordica cochnichinensis* [22], *Benicasa hispida* [23], *C*. *moschata* [24], and *C*. *pepo* [25]. The goals of this study were to determine the novel transcriptional regulatory mechanisms in pumpkin infected with PM for identifying the key genes involved in the defense against PM and to facilitate further understanding of molecular mechanisms against pathogen attack in pumpkin. For this purpose, a time-course transcriptome analysis was employed. Finally, we comprehensively characterized the transcriptomic expression profiles and uncovered novel aspects of the signaling pathway and metabolism in PM-infected plants, including hormone signal transduction, transcription factors and defense against stress. These findings will provide new insights into improving crop resistance.

## Materials and methods

### Plant materials and powdery mildew inoculation

The pumpkin materials (two genotypes of *C*. *moschata*) tested in this study, inbred line “112–2” and cultivar “Jiujiangjiaoding” (abbreviated “JJJD”), which have been shown to be resistant and susceptible to PM, were provided by the Henan Institute of Science and Technology, Xinxiang, Henan, China [26]. The inbred line “112–2” has been purified by more than eight consecutive generations of self-pollination and showed high resistance to PM in an 8-year outdoor field observation study. The seeds were presoaked in warm water (50–60°C) for approximately 20 min to promote germination, after which they were placed on moistened filter paper in a growth chamber (28°C and 60–80% relative humidity in darkness). When at least 80% of the seeds germinated, they were placed in 9-cm-deep plastic pots containing a 1:1 mixture of soil and peat and grown (at day/night temperatures of 28/18°C, a 15 h photoperiod, and 5500 lux light intensity) for 4 weeks before pathogen inoculation.

PM conidia were collected from naturally infected pumpkin leaves in a local greenhouse. One day before inoculation, highly infected leaves were shaken to remove old conidia in order to produce inoculants consisting of vigorous young spores. A spore suspension at 10^6^ spores/ml was made by soaking heavily infected leaves in tap water containing 0.01% Tween-20. When the plants had developed 3–4 fully expanded leaves (4-week-old plants), the pots were divided into two groups: a control group, which was sprayed with distilled water, and a PM treatment group, which was sprayed with freshly prepared spore suspension solution at AM 08:00–9:00; the surface of the seedlings were completely wetted. Leaves from the inbred line “112–2” were collected from both PM-infected and control plants at 24 and 48 hours post inoculation (hpi). At each time point, two young upper leaves from four separate seedlings were collected to form one sample, wrapped with foil, immediately frozen in liquid nitrogen and stored at -80°C until transcriptome analysis. Leaf tissue samples of inbred line “112–2” and of cultivar “JJJD” were collected at 0, 3, 6, 9, 12, 24, 48 and 72 hpi for quantitative RT-PCR (RT-qPCR) verification. The treatments were arranged in a randomized complete block design that consisted of three independent biological replicates.

### Microscopic examination of PM pathogen infection

The third leaves of inoculated and control (non-inoculated) plants of both genotypes were excised at 24 and 48 hpi and processed for microscopy to perform the assays in triplicate. Leaf tissues were cut into small pieces (0.5–1 cm), fixed and decolorized in acetic acid:ethanol (V:V = 1:3), stained with methyl blue:lacticacid:glycerol:distilled water (W:V:V:V = 1:1:1:10) for 24 h and then rinsed with ddH_2_O. For microscopic observations, leaf segments were stored in 50% glycerin and examined under an OlympusBX-43 microscope (Olympus Corporation, Japan)

### RNA sequencing library construction and Illumina sequencing

The total RNA was extracted separately using Trizol reagent (Invitrogen, USA) following the manufacturer’s protocol. The quantity and quality of the total RNA were checked by a NanoDrop 1000 spectrophotometer (Thermo Fisher Scientific Inc., USA) and by resolution on a 1% non-denaturing agarose gel, respectively. The NEBNext^®^ Ultra^™^ RNA Library Prep Kit for Illumina^®^ (NEB, USA) was used for mRNA fragmentation; first-and second-strand cDNA synthesis and 150 bp paired-end reads were generated in accordance with the manufacturer’s recommendations. To select cDNA fragments that were preferentially 150~200 bp in length, the library fragments were purified with an AMPure XP system (Beckman Coulter, USA). The library preparations were sequenced using an Illumina HiSeq^™^ 2000 platform according to the manufacturer’s instructions. All raw data of the pumpkin transcriptome were deposited in the GenBank Short Read Archive (Accession No. SRR5369792).

### De novo assembly and functional annotation

The raw reads were first filtered to obtain high-quality reads and then assembled de novo into contigs using Trinity software [27]. In this step, clean data (clean reads) were obtained by removing reads containing an adaptor and poly (N) sequences, and those of low quality from the raw data. Also, 90% and 85% cut-off scores were used for downstream processing of Q20 or Q30 reads, after which the Q20, Q30, GC content, and sequence duplication level of the clean data were calculated. Subsequently, the contigs were assembled to construct transcripts with paired-end information and were clustered to obtain unigenes. The assembled unigene sequences were aligned by BLASTx to publicly available protein databases including the Nr (NCBI non-redundant protein), Nt, Pfam (Protein family), COG (Clusters of Orthologous Groups), SwissProt, KEGG (Kyoto Encyclopedia of Genes and Genomes) and GO (Gene Ontology) databases. Homology searches against the Nr database were performed by BLASTx with a cut-off E-value of 1e-5. Unigenes having no homologs in the Nr and SwissProt databases were scanned using ESTScan [28].

### Identification and functional annotation of differentially expressed genes (DEGs)

Gene expression levels were estimated by RSEM [29]. For each sample, two biological replicates were sequenced and the correlation coefficients (R^2^) between replicates were calculated using Pearson correlation. Subsequently, the differential expression detection of genes across libraries was analyzed using the DESeq R package (1.10.1) [30]. The P values were adjusted using the Benjamini and Hochberg method [31]. An adjusted P value (padj) <0.05 found by DESeq and |log (fold change)| >1 constituted the threshold to judge the significance of differences in gene expression across libraries. Furthermore, GO enrichment analysis of the DEGs was implemented by the GOseq R package [32], in which the gene length bias was corrected. In addition, after the data correction with the R package, KOBAS software (version 2.0.12) was used to test the statistical enrichment of PM-responsive genes in the KEGG pathway [33].

### Expression analysis of DEGs using RT-qPCR

The total RNA was extracted from the leaves of pumpkin seedlings treated with PM or distilled water for 0, 3, 6, 9, 12, 24, 48 or 72 h as described above. First-strand cDNA synthesis and RT-qPCR were performed as described by Guo et al. [34]. For relative quantification, the 2^–ΔΔCt^ method was used [35]. The *β-actin* gene was used as an internal control, as it has been reported to be a suitable reference gene for normalization of gene expression in pumpkin [36]. Gene-specific primers were designed using ProbeFinder Version 2.44 (http://www.roche-applied-science.com). The primers pecificity was then confirmed by querying each primer sequence against the Phytozome database using BLASTN algorithms (http://www.phytozome.net/search.php?show$=$blast). The primers were presented in S1 Table.

### Statistical analysis

All data are expressed as the mean ± SD of three independent biological replicates (*n* = 3). The data from replicates of the two treatments (112-2-PM and JJJD-PM) were pooled together for one-way analysis of variance (ANOVA), and differences in the mean values of different treatments were determined using the least significant difference (LSD) method. Statistical procedures were performed using the statistical analysis system software (DPS, version 7.55). Values in P≤ 0.05 were considered statistically significant.

## Results

### Evaluation of resistance in *C*. *moschata* genotypes

**Fig 1:**
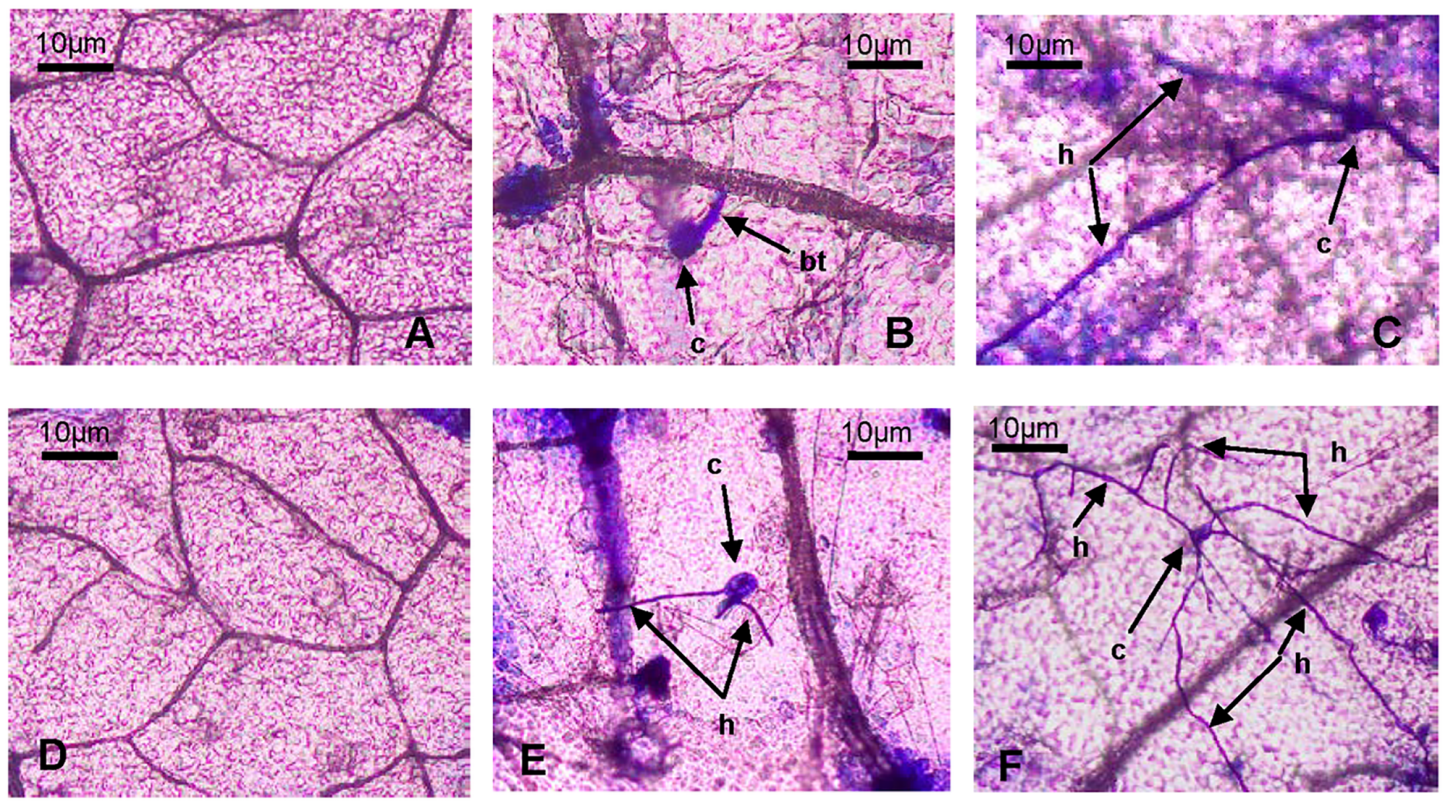
Microscopic observations of powdery mildew infection in *C*. *moschata* genotypes. Non-inoculated leaves of “112–2” (A), 24 h of PM-inoculated leaves of “112–2” (B), 48 h of PM-inoculated leaves of “112–2” (C), Non-inoculated leaves of “JJJD” (D), 24 h of PM -inoculated leaves of “JJJD” (E), and 48 h of PM-inoculated leaves of “JJJD” (F). The arrow indicates the growth of PM pathogen, h, hyphal; c, conidium; bt, bud tube.

Differences in the response to PM between the two genotypes were not yet visible at the time the leaves were sampled for RNA extraction. Fungal growth was cytologically assessed at 24 h and 48 h after inoculation with PM pathogens. Microscopic observations showed no conidia in either of the control genotypes (Fig 1A and 1D). The conidia began to grow bud tubes from the side at 24 hpi, and primary hyphae appeared at 48 hpi on the leaves of “112–2” (Fig 1B and 1C). However, only a few hyphae occurred at 24 hpi on the leaves of “JJJD”; these hyphae bifurcated to form a dense hyphal network (Fig 1E and 1F). These results suggest that the growth speed of PM on the resistant “112–2” genotype was distinctly slower than that on the susceptible “JJJD” phenotype, which may be related to different resistance mechanisms. Hence, we chose to analyze the transcriptome of the PM-resistant inbred line “112–2” during PM infection at 24 h and 48 h.

### Illumina sequencing and de novo assembly

To obtain a global view of the PM-responsive transcriptome of pumpkin leaves, leaves after infection with PM were collected at two time points, 24 h and 48 h, (referred to as PM-L24 and PM-L48, respectively) and at 24 h for control leaves treated with distilled water only (referred to as W-L24). For each sample, two biological replicates were sequenced using an Illumina HiSeq^™^ 2000 platform. The correlation coefficients (R^2^) between replicate samples were 0.702, 0.705 and 0.761 for PM-L241 vs. PM-L242, PM-L481 vs. PM-L482 and W-L241 vs. W-L242 respectively. This finding indicated that there was high similarity among replicate sample selections and that a high-level library existed for subsequent analysis of unigene expression (S1 Fig). After the reads containing adaptors, reads with unknown nucleotides larger than 5% and low-quality reads were removed, at least 17 Gb of clean paired-end reads were generated for each sample (S2 Table). The length distributions of the transcripts and unigenes are shown in S2 Fig. In total, 180793 transcripts (length ≥ 200) with average length of 790 nt were assembled and further generated into 141621 unigenes mean length of which was 591 nt. Among these unigenes, there were 61618 (43.51%) whose size ranged from 300 to 1000 nt and 11639 (8.22%) size of which varied from 1000 to 2000 nt. The length of the assembled unigenes is comparable to the length previously reported in the transcriptome analyses of *C*. *pepo* and *C*. *moschata* [24, 37].

### Identification of DEGs

**Fig 2:**
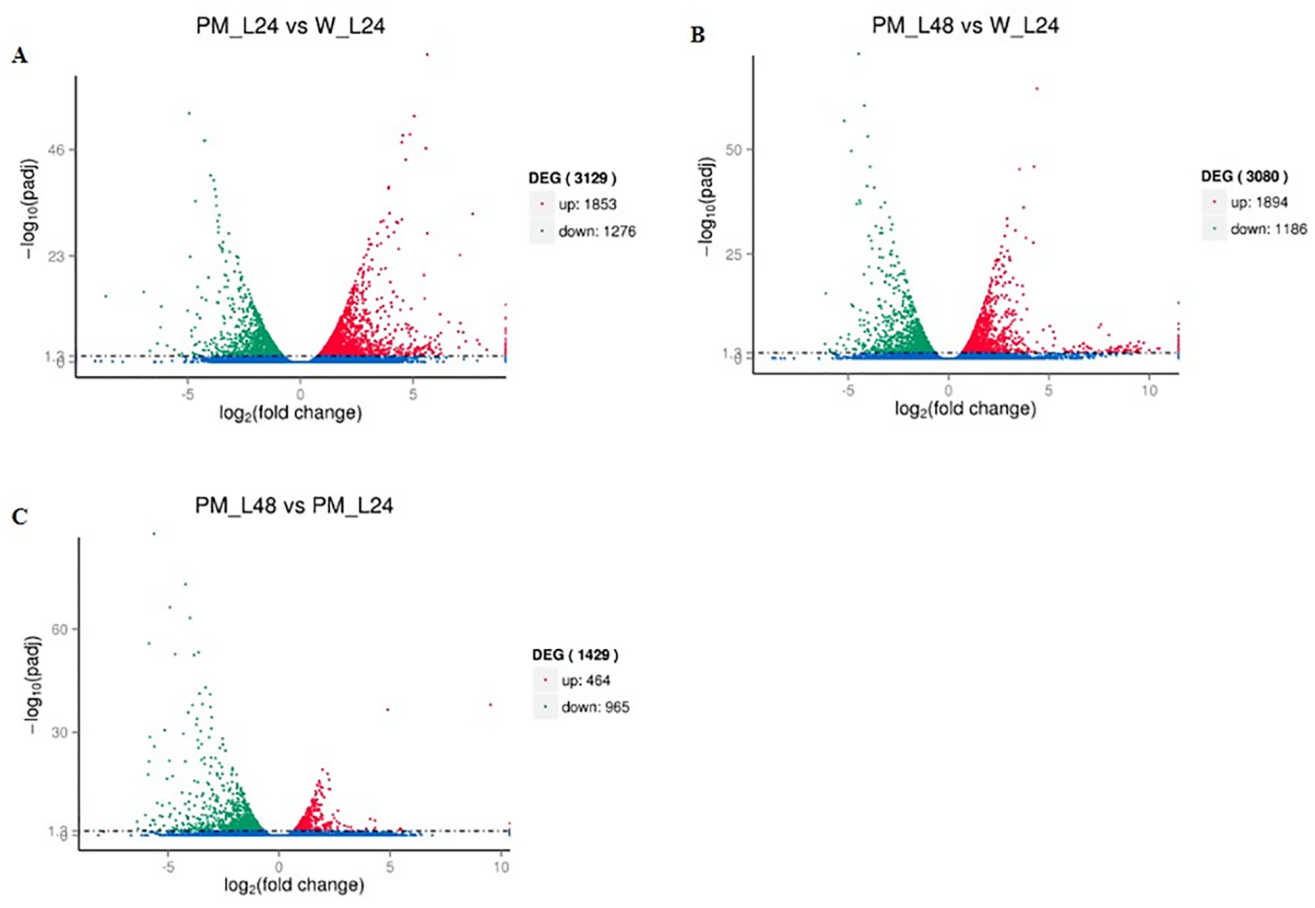
Analysis of unigene expression difference in pumpkin. The x-axis represents the unigene expression fold changes between any two libraries; the y-axis represents the statistically significant analysis of unigene expression differences. The smaller the adjusted call (padj), the greater and more significant the -log10 (padj). The scattered points represent each gene. No significant difference is indicated by blue color, whereas significant up-regulation and down-regulation are indicated by red and green colors, respectively.

The transcription levels were calculated usingthe expected number of fragments per kilobase of transcript sequence per million base pairs sequenced (FPKM) method. The normalized expression levels in PM-inoculated and untreated control plants were compared to detect DEGs. The analysis of up- and down-regulated DEGs by scatterplot is shown in Fig 2 and S3 Table. In total, the expression of 7638 DEGs was detected in the tested samples. Amounts of 3129, 3080 and 1429 DEGs were found in the PM-L24 vs. W-L24, PM-L48 vs. W-L24 and PM-L48 vs. PM-L24 comparisons, respectively. Of these DEGs, 1853, 1894 and 464 were up-regulated in the PM-L24 vs. W-L24, PM-L48 vs. W-L24 and PM-L48 vs. PM-L24 comparisons, respectively. A Venn diagram was constructed to show the number of uniquely expressed transcripts at the two PM response stages (S3 Fig). A total of 2767 DEGs were sample-specific. Amounts of 1089, 1256 and 422 DEGs were specifically expressed in the PM-L24 vs. W-L24, PM-L48 vs. W-L24 and PM-L48 vs. PM-L24 comparisons, respectively. A total of 4716 DEGs were expressed both in the PM-L24 vs. W-L24 and PM-L48 vs. W-L24 comparisons, and 129 DEGs were expressed in all three comparisons. To validate the gene expression levels determined by the RNA-Seq data, 16 representative unigenes were evaluated by RT-qPCR analysis in a separate experiment. The results from the RT-qPCR and RNA-Seq analyses of these genes were then compared. The strong correlation (R^2^ = 0.832 and 0.851) between the RNA-Seq and RT-qPCR expression values both at 24 hpi and 48 hpi validates the RNA-Seq data (S4 Fig).

### GO terms and KEGG pathway annotation of DEGs

**Fig 3:**
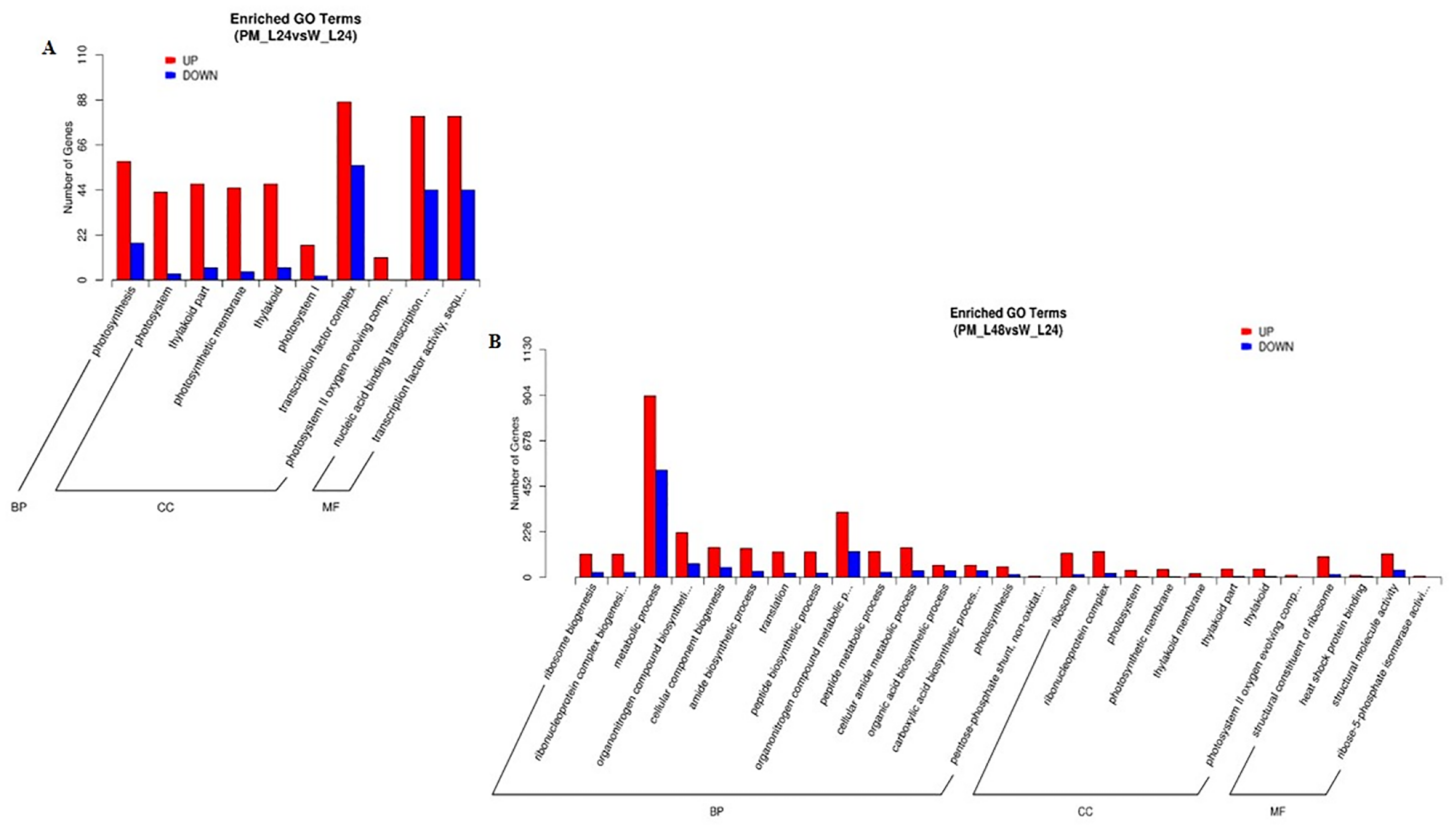
Enriched GO terms of DEGs. A, DEGs at 24 h after PM inoculation compared with untreated control plants; B, DEGs at 48 h after PM inoculation compared with untreated control plants. The y-axis indicates the number of genes in a subcategory, and the x-axis indicates the different subcategories.

To further establish the main function of the unigenes involved in the response to pumpkin inoculation with PM, functional classifications were defined using GO terms from the GO database (http://www.geneontology.org/). This approach provided broad functional classifications regarding the three major GO functional domains (biological processes, cellular components and molecular functions). There were 2039 DEGs categorized into one or more GO terms that consisted of 1 biological process, 7 cellular components and 2 molecular function subcategories in the PM-L24 vs. W-L24 comparison (Fig 3A). Among these terms, photosynthesis (GO:0015979) was enriched in the biological process category, implying that marked changes occurred in the expression of genes involved in photosynthesis of pumpkin seedlings at 24 hpi. In addition, a GO term was considered significantly enriched if the false discovery rate was below 0.05. Seventy DEGs were found to be significantly enriched in photosynthesis, with 55 up- and 17 down-regulated (S4 Table). DEGs coding for the photosystem I reaction center subunit (psaK), photosystem II reaction center W protein (psbW), photosystem I reaction center subunit III (psaF), PSII 5 kDa protein, and L-type lectin-domain-containing receptor kinase were significantly up-regulated by ~2 fold at 24 hpi and plastidic glucose transporter 1 and MADS-box transcription factor 23 were significantly down-regulated by ~2 fold both at 24 hpi and 48 hpi. Furthermore, a total of 2178 DEGs were categorized into functional groups consisting of 16 biological processes, 8 cellular components and 4 molecular function subcategories in the PM-L48 vs. W-L24 comparison (Fig 3B). Among the 16 biological processes, the predominant categories were metabolic process (GO: 0008152, 65.7%) and organonitrogen compound biosynthetic process (GO: 1901564, 20.6%). There were 157 DEGs that were significantly enriched in metabolic process, with 94 up- and 63 down-regulated (S5 Table). Genes coding for acyl-CoA synthetase and the tyrosine-protein kinase FRK-like isoform were significantly down-regulated by ~2 fold at 48 hpi.

Many DEGs encoding for the photosystem I and II reaction centers, oxygen-evolving enhancer protein, chlorophyll A/B binding protein, and magnesium chelatase (Chl synthesis-related gene) were found to be up-regulated both at 24 hpi and 48 hpi. The up-regulation of these genes suggested that the photosynthetic apparatus was not undergoing degradation after 48 h of infection and hence not enough to lead to a reduction in the photosynthetic rate, maybe associated with that the growth stage of pathogens, which coincides with the appearance of primary hyphae (Fig 1C). These results in this study were not in agreement with the statement of a previous report, according to which the expression profiles of many proteins involved in photosynthesis were mostly down-regulated in PM infected wheat leaves [38]. Interestingly, two DEGs encoding the chloroplastic ATP synthesis-related protein (c47457_g1 and c115085_g1) were up-regulated both at 24 hpi and 48 hpi. This protein catalyzes the synthesis of ATP, probably because plants need more energy to activate various defense responses during PM-pumpkin interactions.

Pathway definitions were derived from the KEGG (Kyoto Encyclopedia of Genes and Genomes) database (S6 Table). Ten significantly enriched pathways with a corrected P value of less than 0.05 related to PM inoculation were identified in the PM-L24 vs. W-L24 analysis; 7 and 11 significantly enriched pathways were identified in the PM-L48 vs. W-L24 and PM-L48 vs. PM-L24 analyses. Among these significantly enriched pathways, 3 pathways, including plant hormone signal transduction (ko04075), carotenoid biosynthesis (ko00906), porphyrin and chlorophyll metabolism (ko00860), were common to three comparisons. In addition, photosynthesis (ko00195) was common to both the PM-L24 vs. W-L24 and PM-L48 vs. W-L24 comparisons.

### DEGs involved in hormone signal transduction pathway

Plant resistance to pathogens depends on the interplay of different signaling mechanisms, such as those mediated by the hormones salicylic acid (SA), jasmonic acid (JA), and ethylene (ET) [39]. In general, SA is required to defend against biotrophic pathogens that benefit from a live host cell, while JA and ET are effective against necrotrophs that benefit from host cell death [40]. In addition to these well characterized pathways, other plant hormones, such as abscisic acid (ABA) and auxins, are emerging as important coregulators of plant resistance to pathogens [41]. In this study, a total of 58 DEGs, e.g., AUX1, AUX/IAA, TIR1, CRE1, SAUR, PYR/PYL, SnRK2, EIN, ERF1, TGA, MYC2, among others, were identified that showed high similarity to many genes related to plant hormone signaling pathways (S7 Table). In detail, 49 DEGs (39 up-regulated and 10 down-regulated), 35 DEGs (23 up-regulated and 12 down-regulated) and 11 DEGs (4 up-regulated and 7 down-regulated) were identified in the PM-L24 vs. W-L24, PM-L48 vs. W-L24 and PM-L48 vs. PM-L24 comparisons, respectively. Of these DEGs, only 28 (20 up-regulated and 7 down-regulated) were commonly regulated both in the PM-L24 vs. W-L24 and PM-L48 vs. W-L24 comparisons, implying that these DEGs might play major roles in resistance to PM in pumpkin.

Six AUX1, 3 TIR, 7 AUX/IAA and 10 SAUR genes involved in auxin signaling were identified to be up-regulated in PM-L24 vs. W-L24. Similarly, 2 AUX1, 1 TIR, 3 AUX/IAA and 6 SAUR genes were found to be up-regulated in the PM-L48 vs. W-L24 comparison. These results were not consistent with previous reports describing that the expression of AUX/IAA (auxin/indole-3-acetic acid) is repressed in response to PM (*E*. *pisi*) in resistant *Medicago truncatula* [17]. TGA transcription factors have been suggested to be important regulators of SA-mediated pathogen resistance and negatively regulate the expression of pathogenesis-related genes such as PR1 [42]. One (c8347_g1) TGA gene (3) was down-regulated both at 24 and 48hpi. Two ABHD (abscisic acid 8'-hydroxylase) and 2 NCED (9-cis-epoxycarotenoid dioxygenase) genes involved in ABA biosynthesis were identified to be down-regulated both at 24 hpi and 48 hpi. In addition, 4 up-regulated PYR/PYL genes, 4 down-regulated PP2C (5) genes and 1 up-regulated SnRK2 gene were identified at 24 hpi or 48 hpi, suggesting that ABA signaling might play a vital role in pumpkin seedling responses to PM. ABA treatment increases the resistance of barley against PM [43], and repression of ABA biosynthesis is associated with PM penetration resistance of non-host *Arabidopsis* [44]. Activated ABA receptors have been demonstrated to be formed by an ABA-binding RCAR/PYR1/PYL family member and the receptor blocks the phosphatase activity of PP2Cs; consequently, protein kinases such as SnRKs are no longer inhibited, and they phosphorylate key targets of the ABA signaling pathway [45, 46].

### Differential expression of transcription factor transcripts

Transcriptional regulation of plant genes is a central step in plant defense responses. Therefore, elucidation of the complex regulatory mechanisms that control defense gene expression among plant species is important for understanding the molecular basis of plant–pathogen interactions. Increasing numbers of transcription factors (TFs), including members of the WRKY, NAC, bHLH, bZIP, ERF/AP2, and MYB families, have been reported to play crucial roles in plant defense against pathogen attack [17, 47]. In this study, 180 TFs were identified based on their assigned protein families, including WRKY, MYB (MYB domain), HSF (heat stress transcription factor A), MADS (MADS-box), HD-ZIP (homeobox-leucine zipper), and bHLH (basic helix-loop-helix) and ERF (Ethylene response factor) TFs (S8 Table). These results were consistent with previously reported TFs of other plants after inoculation with PM [17, 20]. In detail, 143 DEGs (93 up-regulated and 50 down-regulated), 95 DEGs (54 up-regulated and 41 down-regulated) and 49 DEGs (16 up-regulated and 33 down-regulated) were identified in the PM-L24 vs. W-L24, PM-L48 vs. W-L24 and PM-L48 vs. PM-L24 comparison, respectively. Of these DEGs, only 58 (30 up-regulated and 27 down-regulated) were commonly regulated both in the PM-L24 vs. W-L24 and PM-L48 vs. W-L24 comparisons, whereas 24 TFs (15 up-regulated and 9 down-regulated) were regulated in PM-L48 vs. W-L24 only. Remarkably, bHLHs (33), ERFs (23), WRKYs (11), HD-ZIPs (9), and HSFs (7) were the most frequently identified as being up or down-regulated in three comparisons, implying that they might regulate resistance to PM. Among these TFs, 6 bHLHs, 4 ERFs and 1 WRKY were up-regulated and 4 HSFs, 2 bHLHs, 3 ERFs, 2 HD-ZIPs and 1 WRKY were down-regulated; these up- and down-regulated TFs were common to both the PM-L24 vs. W-L24 and PM-L48 vs. W-L24 comparisons.

### Differential expression of stress/defense transcripts

Plant defense responses are involved in defense response gene activation upon pathogen infection. In this study, some unigenes were identified to be involved in the defense response to PM in pumpkin seedlings (S9 Table). Two orthologs of the *Cucumis sativus MLO3* gene (c63983_g1) and the *Cucumis melo MLO3* gene (c134562_g1) were identified to be down-regulated by ~2.33 fold both at 24 hpi and 48 hpi. Another *SGT1* homolog (c8328_g1) in our findings was also down-regulated both at 24 hpi and 48 hpi. Reactive oxygen species (ROS) are associated with the hypersensitive response [48], which is related to program cell death and plays a critical role in resistance to PM [16]. Some DEGs encoding antioxidant enzymes were common to both the PM-L24 vs. W-L24 and PM-L48 vs. W-L24 comparisons, such as 11 peroxidases (3 up-regulated and 1 down-regulated), ascorbate peroxidase (1 up-regulated and 2 down-regulated) and superoxide dismutase (1 down-regulated). These results were in agreement with those of recent studies [16, 17], in which enzymes involved in ROS metabolism, such as peroxidase, were regulated in plants after PM inoculation.

### Confirmation of PM-regulated DEGs

**Fig 4:**
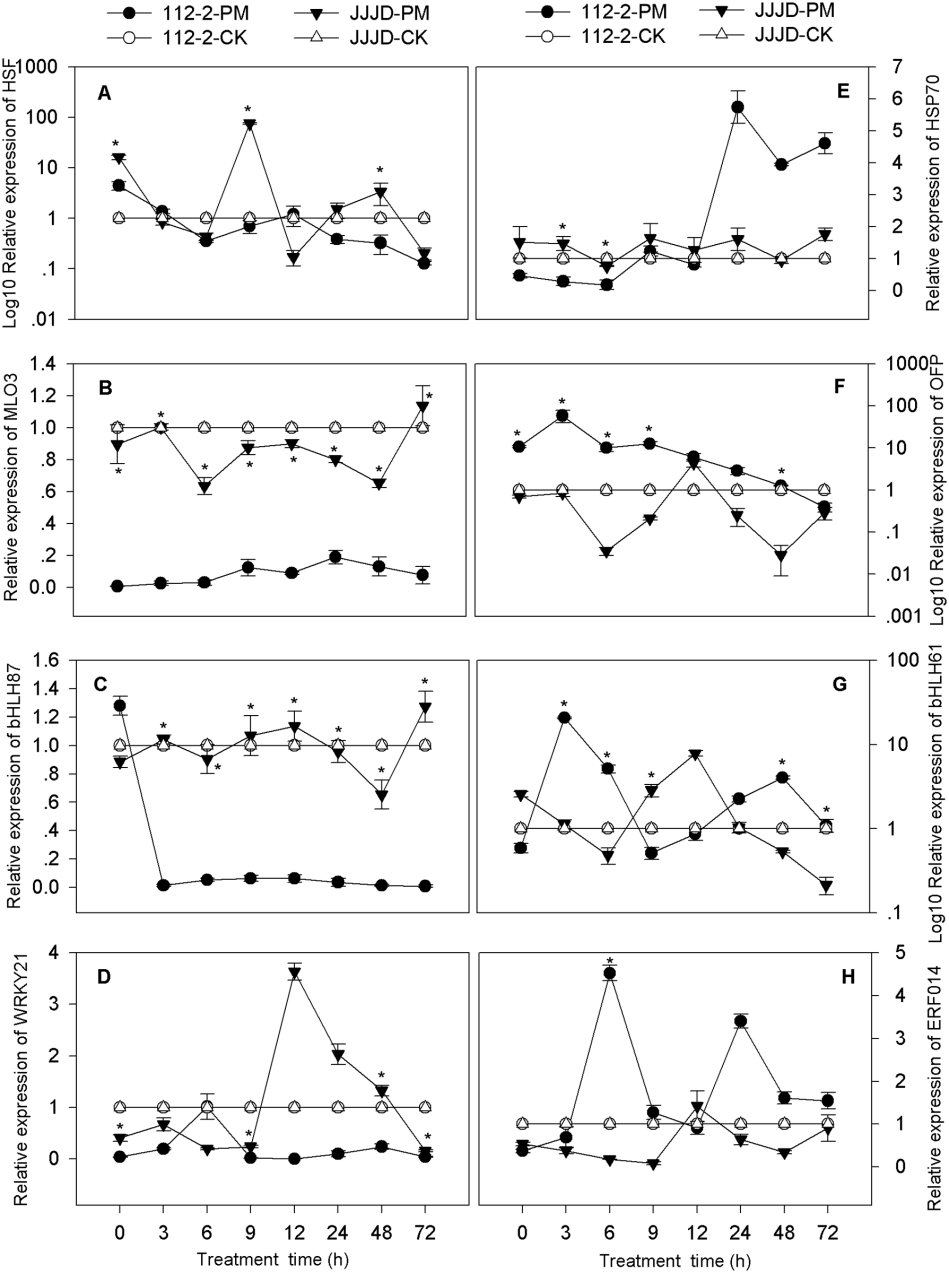
Analysis of the mRNA expression of 8 genes using RT-qPCR in PM-resistant and PM-susceptible plants. Total RNA was extracted from pumpkin leaves that were sprayed with a spore suspension or water. The pumpkin *β-actin* gene was used as an internal reference gene. The expression levels of the genes of plants sprayed with water only at each time point were used as controls. The relative gene expression in A, F and G (Y-axis) was transformed to a log_10_ scale. The values are the means ± SEs of three biological replicates.*indicates significant differences (P<0.05).

**Fig 5:**
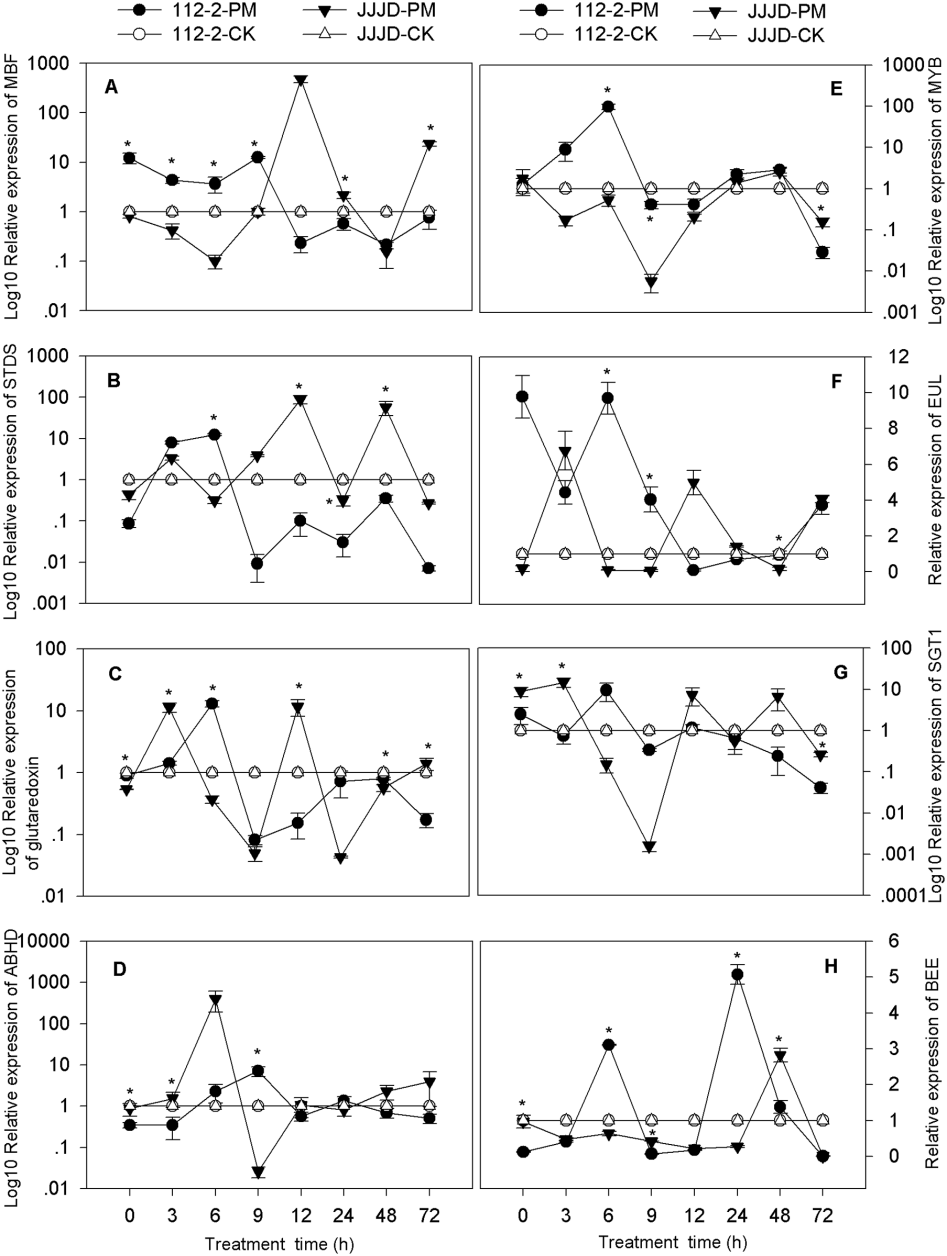
Analysis of the mRNA expression of other 8 genes using RT-qPCR in PM-resistant and PM-susceptible plants. Total RNA was extracted from pumpkin leaves that were sprayed with a spore suspension or water. The pumpkin *β-actin* gene was used as an internal reference gene. The expression levels of the genes of plants sprayed with water only at each time point were used as controls. The relative gene expression in A, B, C, D, E and G (Y-axis) was transformed to a log_10_ scale. The values are the means ± SEs of three biological replicates.*indicates significant differences (P<0.05).

To verify that the genes identified from the transcriptome sequencing libraries were differentially expressed, RT-qPCR analysis was performed to confirm the expression profiles of 16 selected unigenes that were up- or down-regulated by PM inoculation. The transcript levels of these unigenes were investigated at different time points (0, 3, 6, 9, 12, 24, 48 and 72 hpi) using resistant “112–2” and the susceptible cultivar “JJJD” as test materials and were compared with those of the water-sprayed control treatments (Figs 4 and 5). All the data were normalized to that of the *β-actin* gene and were related to the transcripts of corresponding unigenes in water-sprayed control plants at each time point.

The transcripts of HSF (c72139_g2), protein MLO3 (c63983_g1), bHLH87 (c66236_g1) and WRKY21 (c117900_g1) in the 112-2-PM treatment showed complete down-regulation during the whole infection period compared with thetranscripts in the water treatment (Fig 4A, 4B, 4C and 4D). The transcript levels of these four DEGs in the 112-2-PM treatment were essentially lower than those in the JJJD-PM treatment, and the different expression of HSF and WRKY21 was significant at 0, 12, 9 and 48 hpi; the expression of MLO3 and bHLH87 was also significant during the whole infection period. The expression of hsp70 (heat shock protein 70, c115600_g1) in the 112-2-PM treatment was dramatically lower than that in the JJJD-PM treatment both at 3 and 6 hpi but was higher after 24 hpi (Fig 4E). The OFP (c119105_g1) and ERF014 (c146381_g1) transcripts in the 112-2-PM treatment were up-regulated by PM during the whole infection time (Fig 4F and 4H). The expression of OFP and ERF014 in the 112-2-PM treatment was higher than that in the JJJD-PM treatment; significant differences were especially observed before 9 hpi and 6 hpi, respectively. The expression of MBF (multiprotein-bridging factor, c101829_g1) and STDS (strictosidine synthase, c60148_g2) was significantly higher before 9 hpi and 6 hpi in the 112-2-PM treatment than in JJJD-PM treatment, clearly lower thereafter (Fig 5A and 5B). The expression of bHLH61 (c71304_g1), glutaredoxin (c143537_g1) and BEE (c68108_g2) in the 112-2-PM treatment dramatically changed compared with that of the JJJD-PM treatment (Figs 4G, 5C and 5H). The expression of ABHD (abscisic acid hydroxylase, c71433_g2) was generally lower in the 112-2-PM treatment than in the JJJD-PM treatment during the whole period (except for 9hpi), and significant differences were especially observed at 3hpi (Fig 5D). The transcript levels of MYB (c142659_g1) and EUL (ubiquitin-protein ligase, c44996_g1) both at 6 and 9 hpi were significantly higher in the 112-2-PM treatment than in the JJJD-PM treatment (Fig 5E and 5F). The expression of SGT1 (c8328_g1) at 3 hpi was significantly lower in the 112-2-PM treatment than in the JJJD-PM treatment, slightly higher at 6 and 9 hpi, and again lower thereafter (Fig 5G).

## Discussion

Much attention has been paid to the nutritional and pharmacological properties of *C*. *moschata*. Cucurbit powdery mildew is a serious biotrophic pathogen disease in field and greenhouse cucurbit crop worldwide. Recently, RNA-Seq technology has been used to study the gene expression of members of the Cucurbitaceae family [6–8]. However, no study on the comprehensive identification of the DEGs during PM inoculation has been conducted in *C*. *moschata* to date. In this study, using RNA-Seq technology, 4716 DEGs were identified to be involved in the response to PM of pumpkin. To our knowledge, this is the first study to investigate transcriptional changes underlying the resistance mechanism. Moreover, the results in this study implied that these DEGs are likely to compose a pool of candidates for breeding for PM resistance.

HSFs are known to play dominant roles in plant responses to biotic stress stimuli. The disease resistance of the *Arabidopsis* mutant *hsfB2b* to *Alternaria brassicicola* improved significantly [49] and overexpression of *VpHsf1* in tobacco exhibited enhanced susceptibility to *Phytophthora parasitica* [50]. In this study, four HSFs (c72757_g3, c115757_g1, c72139_g2, c59697_g1) were found to be down-regulated both at 24 hpi and 48 hpi (S8 Table), and the expression of one (c72139_g2) of these HSFs in the resistant genotype was significantly lower than that in the susceptible genotype during the 72 h of infection (Fig 4A), implying that this HSF gene is a candidate gene and plays role in the negative regulation of resistance to PM in pumpkin. Previous studies have shown that WRKY members are involved in the regulation of gene-mediated disease resistance as well as in the regulation of transcriptional reprograming associated with plant immune responses [47, 51]. In this study, 11 WRKYs were up or down-regulated in three comparisons, including 6 that were up-regulated at 24 hpi as well as 3 up- and 3 down-regulated WRKY genes at 48 hpi (S8 Table). These results were in accordance with the results of recent studies in that WRKY TFs are involved in the defense of *Cucumis sativus* to PM [52]. One of these WRKYs (WRKY 21) showed significantly lower expression in the 112-2-PM treatment than in the JJJD-PM treatment at the late stage (48 and 72 hpi) of infection (Fig 4E), supporting the critical role of this TF family in the plant defense response against fungal pathogens. Most WRKY TFs are negative regulators of defense responses in plant. For example, 12 *FvWRKY* genes from strawberry were down-regulated during PM infection [53]. Transgenic *AtWRKY48*-overexpressing plants showed enhanced susceptibility while the loss-of-function *AtWRKY48* mutants showed enhanced resistance to *Pseudomonas syringae* [54]. The bHLH TFs up-regulated by PM are involved in regulating the expression of JA-responsive genes [55], products of which mediate the transcriptional reprogramming associated with the plant immune response. Overexpression of *TabHLH060* has been demonstrated to enhance the susceptibility of transgenic *Arabidopsis* to *Pseudomonas syringae* [56]. Thirty-three bHLHs that regulate pumpkin-PM interactions were identified, including 6 that were up-regulated and 2 that were down-regulated, both at 24 hpi and 48 hpi (S8 Table). One of them (bHLH87) was significantly lower in the resistant genotype than in the susceptible genotype during the period of 72 hpi (Fig 4D), implying that this unigene negatively regulated the resistance to PM in pumpkin. ERF in wheat positively regulates the defense responses to necrotrophic pathogens by activating defense- and stress-related genes that are downstream of the ET signaling pathway [57]. Overexpression of *VpERF2* and *VpERF3* in tobacco has been demonstrated to enhance resistance to PM [58]. Twenty-three DEGs encoding ERFs were identified in three comparisons, including 4 that were up-regulated and 3 that were down-regulated both at 24 hpi and 48 hpi in “112–2” (S8 Table). The expression of ERF014 in the 112-2-PM treatment was higher than that in the JJJD-PM treatment, significant differences were observed especially at the early stage (6 hpi) of infection (Fig 4H), suggesting that this gene positively regulates the response to PM in pumpkin seedlings.

*MLO* (Mildew Locus O) is a plant-specific gene family, whose members are known to respond to biotic stress in various plant species. For example, *CsaMLO8* in cucumber has been characterized as a functional susceptibility gene to PM, particularly in the hypocotyls where it is transcriptionally up-regulated upon inoculation with PM [19]. Two DEGs encoding the MLO3 protein were found to be down-regulated both at 24 hpi and 48 hpi and involved in the defense response according to their GO annotation (S9 Table). In addition, the expression of the MLO3 (c63983_g1) protein in the 112-2-PM treatment was significantly lower than that in the JJJD-PM treatment for 72 hpi (Fig 4B). Therefore, this gene was considered a negative candidate, associated with the resistance of PM in pumpkin. The SGT1 protein (Suppressor of G-Two Allele of Skp1) is essential for protein-mediated resistance in many plant species. The overexpression of *Hv-SGT1* in wheat was demonstrated to enhance the resistance to PM, which was correlated with increased levels of whole-cell ROS at the sites of penetration by the pathogens [59]. The expression change of SGT1 (c8328_g1) in the PM-infected resistant genotype was significantly different from that of the PM-infected susceptible genotype (Fig 5G), maybe associated with the resistance of PM in pumpkin.

## Conclusions

In this research, we used RNA-Seq to study the transcriptome of an important non-model crop species in response to PM. According to the data, we speculated that regulatory networks including hormone signal transduction pathways, TFs and defense-response metabolism control the expression of genes involved in resistance to PM in *C*. *moschata*. In addition, 6 unigenes of the 16 DEGs confirmed using RT-qPCR showed significant differences in the resistant inbred line “112–2” compared to the susceptible material, and these unigenes may represent promising candidates that are involved in regulating the defense response (bHLH87, ERF014, WRKY21, HSF, MLO3, and SGT1).

## Supporting information

S1 Fig The correlation values between replicate samples. The x-axis is the fragments per kilobase of transcript sequence per millions base pairs (FPKM) + 1-fold-change log_10_values of sample 1; the y-axis is plotted against the FPKM+1-fold change log_10_values. R^2^: Pearson squared correlation coefficients.
(DOC)

S2 Fig Length distribution of transcripts and unigenes. (DOC)

S3 Fig Venn diagram showing the distribution of DEGs. (DOC)

S4 Fig Comparison of transcription levels measured by RNA sequencing (RNA-Seq) and quantitative RT-PCR (qRT-PCR) assays. The gene expression values were transformed to the log_10_ scale. The FPKM-fold-changes log_2_values (x-axis) were plotted against the qRT-PCR fold-change log_10_values (y-axis). Pumpkin *β-actin* was used as an internal control to normalize the expression data. Each value denotes the mean relative level of expression of three biological replicates.
(TIFF)

S1 Table The sequence of primers employed in this study. (DOC)

S2 Table Output of the transcriptome sequencing for pumpkin. (DOC)

S3 Table Genes differentially expressed in powdery mildew infected pumpkin. (XLSX)

S4 Table List of PM responsive DEGs in photosysthesis in three comparisons. (XLS)

S5 Table List of PM responsive DEGs in metabolic processes in three comparisons. (XLS)

S6 Table List of enriched pathways for DEGs in three libraries based on pairwise comparison. (XLS)

S7 Table List of DEGs of hormone signal transduction pathways in three comparisons. (XLS)

S8 Table List of PM responsive TFs in three comparisons. (XLS)

S9 Table List of PM-responsive defense stresses in three comparisons. (XLS)
