# Supplementary figures and images for: Transcriptome profiling of pumpkin (Cucurbita moschata Duch.) leaves infected with powdery mildew
Source: PLoS One. 2018 Jan 10;13(1):e0190175. doi: 10.1371/journal.pone.0190175 (PMC5761878; doi:10.1371/journal.pone.0190175)

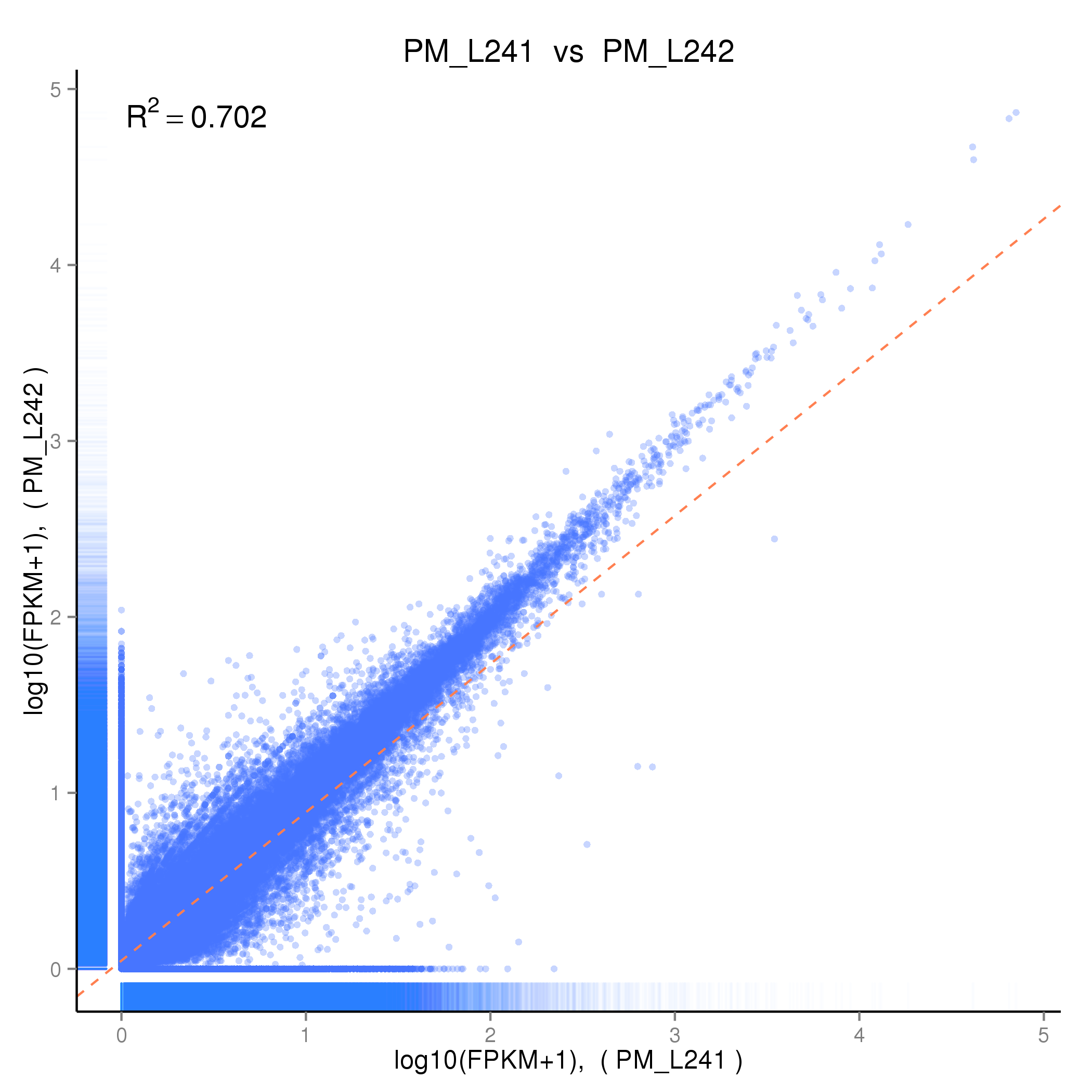

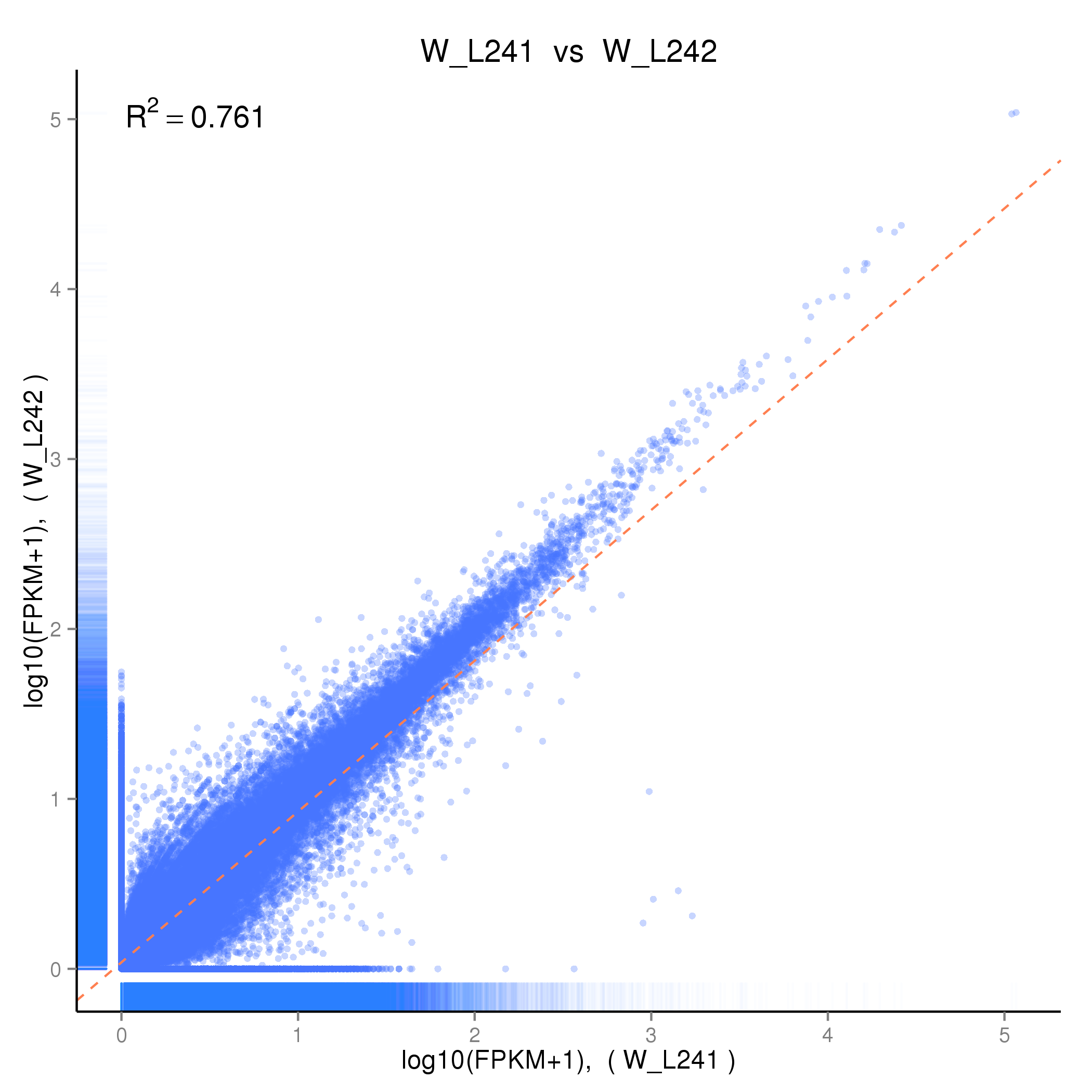

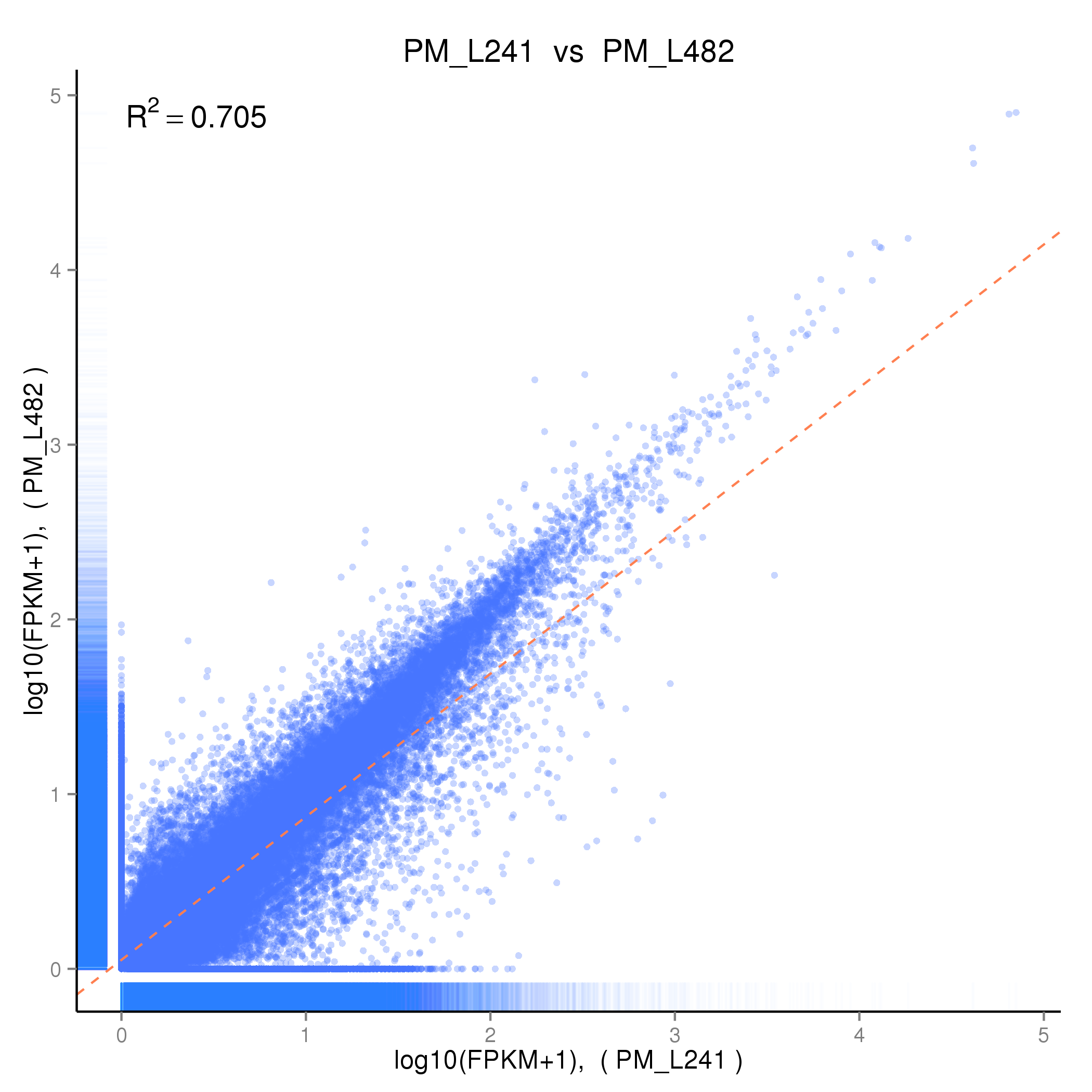

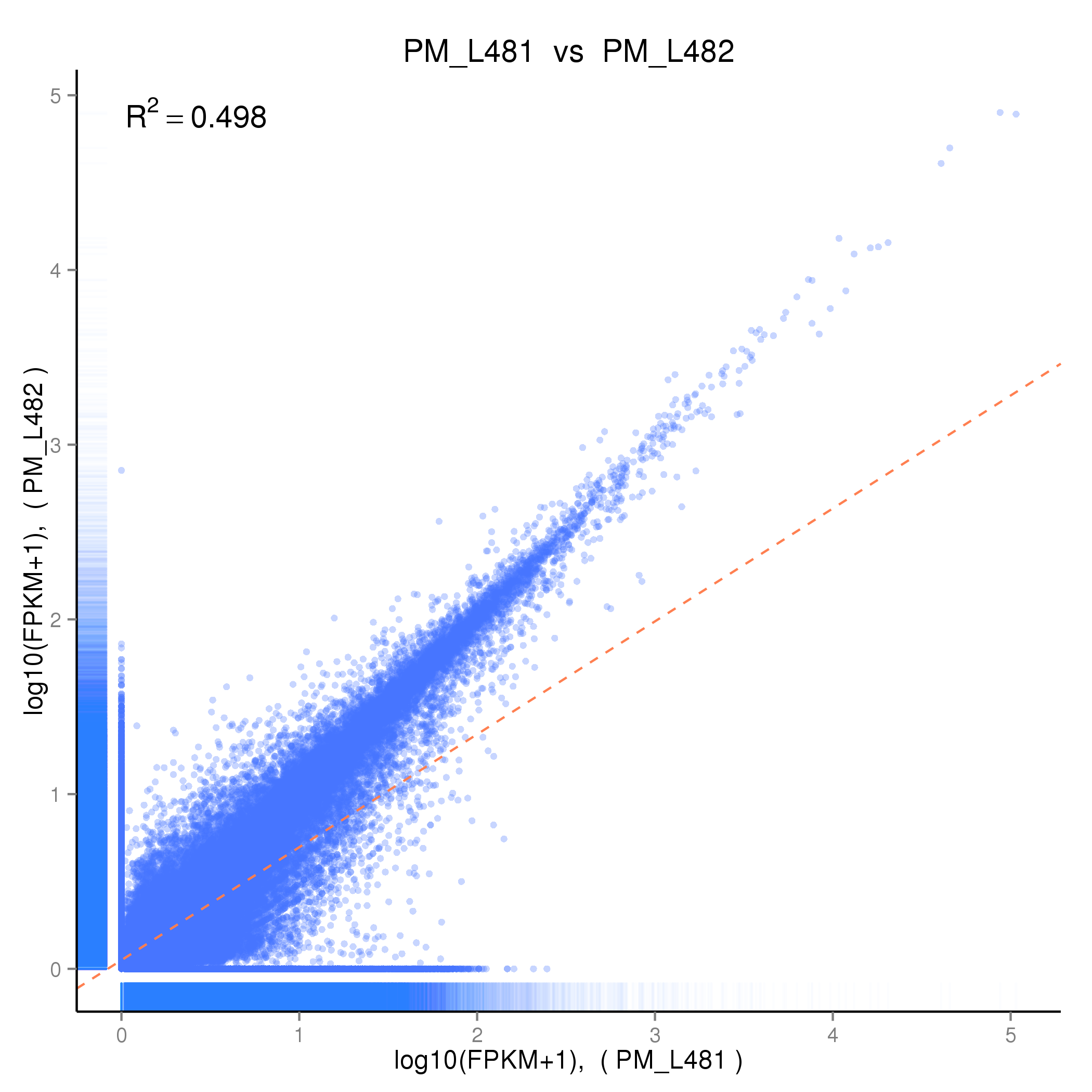

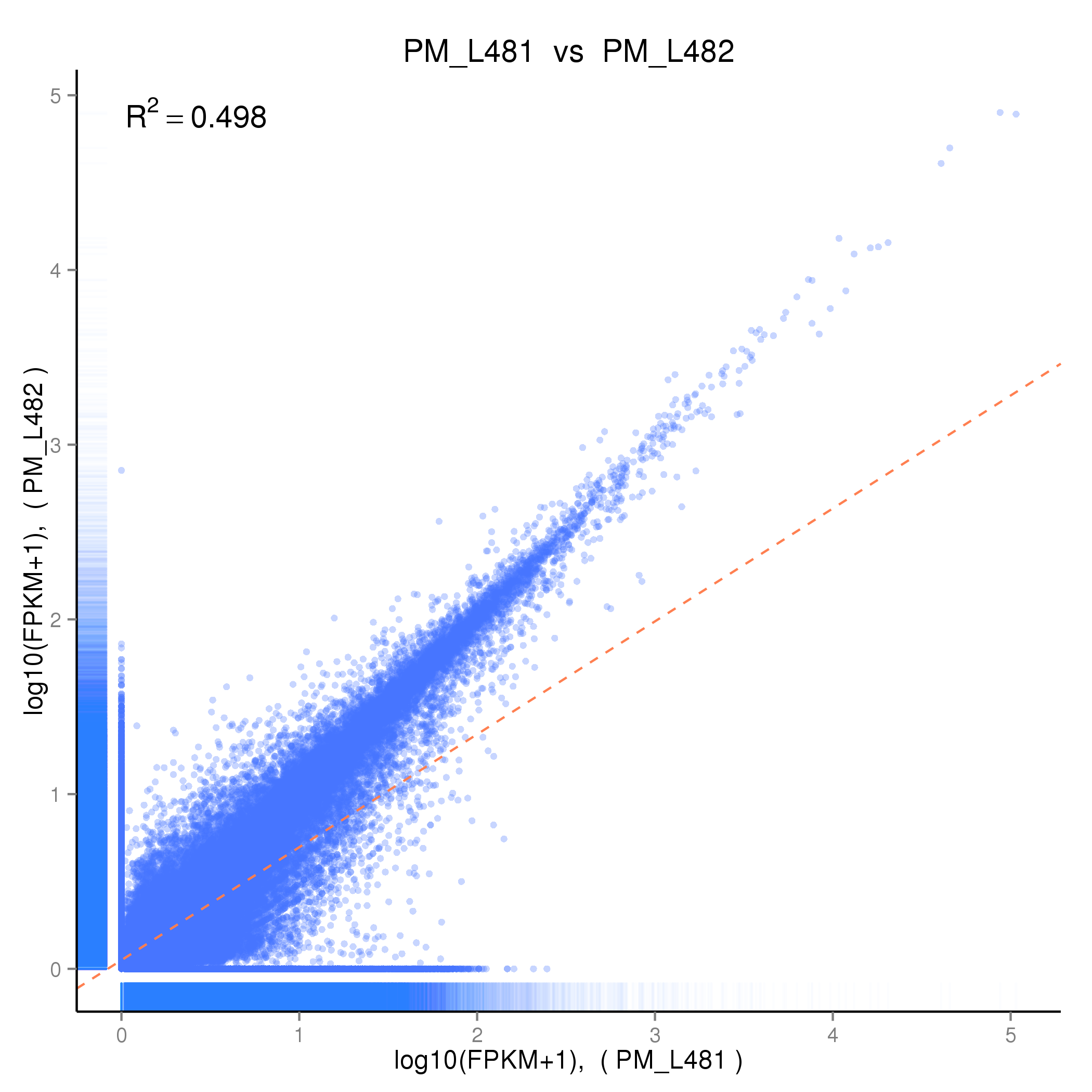


A

B

C

Supplement: S1 Fig — The x-axis is the fragments per kilobase of transcript sequence per millions base pairs (FPKM) + 1-fold-change log10values of sample 1; the y-axis is plotted against the FPKM+1-fold change log10values. R2: Pearson squared correlation coefficients. (DOC) [file pone.0190175.s001.doc]

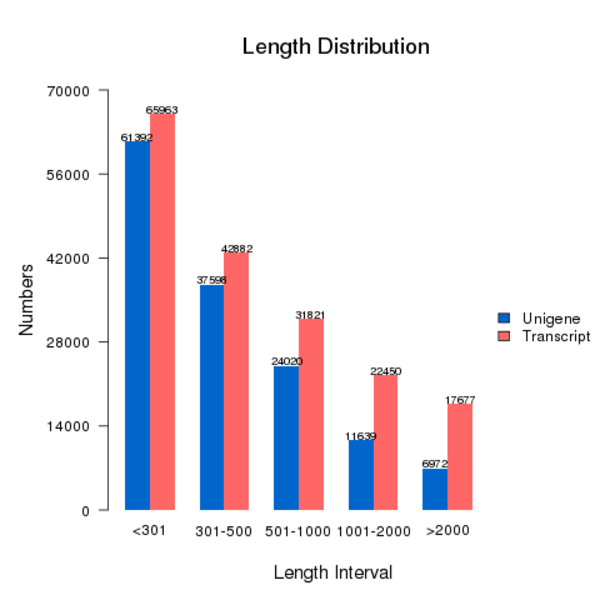

Supplement: S2 Fig — (DOC) [file pone.0190175.s002.doc]

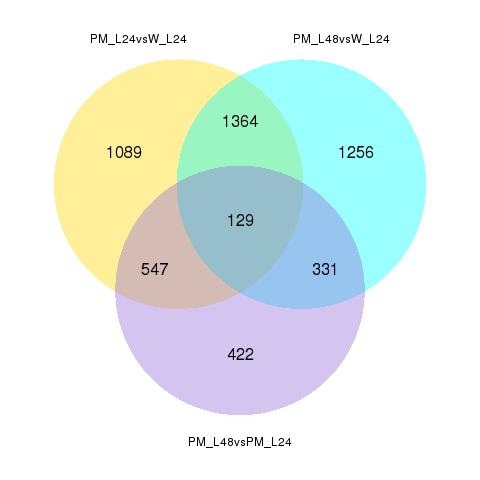

Supplement: S3 Fig — (DOC) [file pone.0190175.s003.doc]

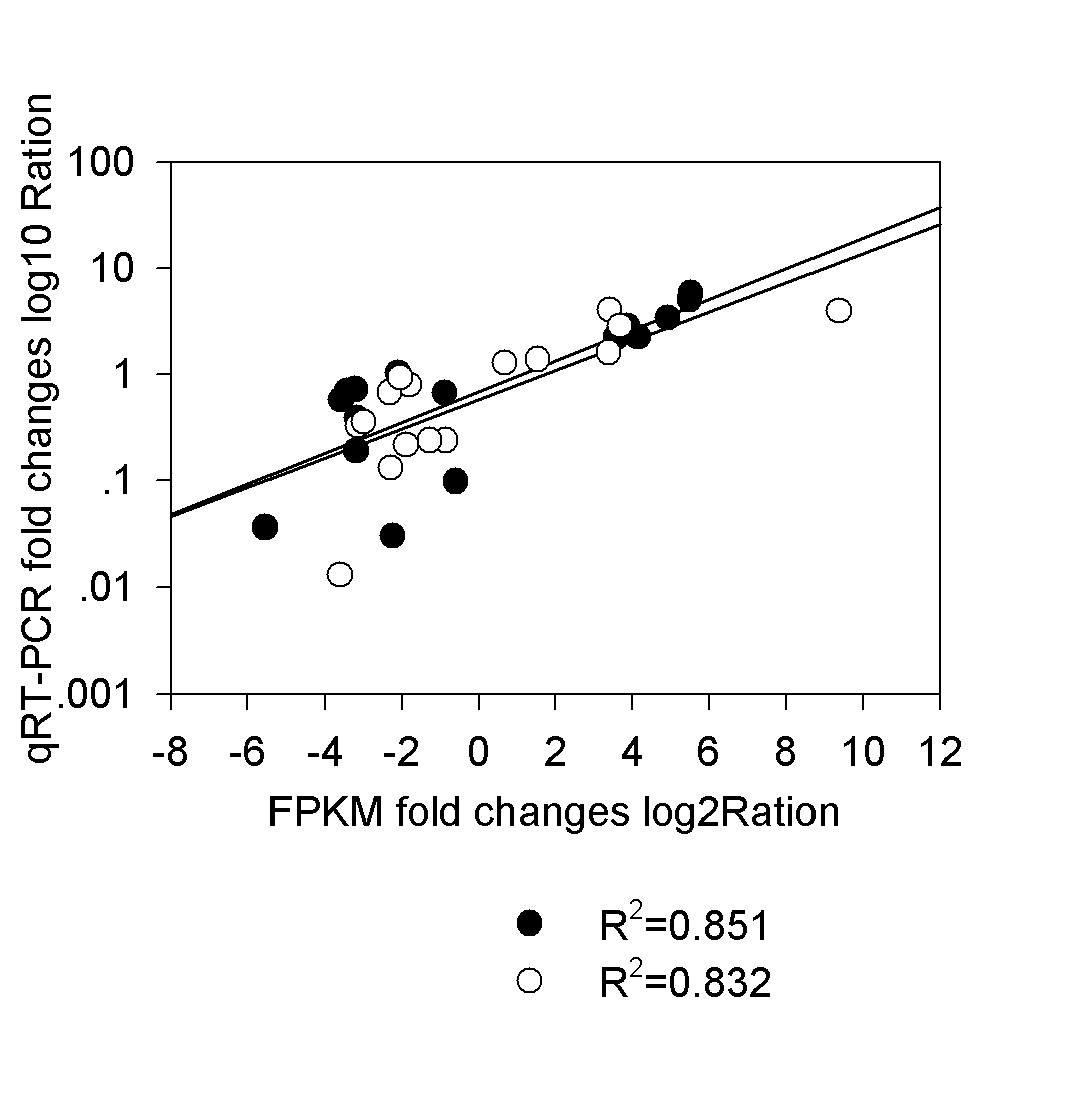

Supplement: S4 Fig — The gene expression values were transformed to the log10 scale. The FPKM-fold-changes log2values (x-axis) were plotted against the qRT-PCR fold-change log10values (y-axis). Pumpkin β-actin was used as an internal control to normalize the expression data. Each value denotes the mean relative level of expression of three biological replicates. (TIFF) [file pone.0190175.s004.tiff]
