# Supplementary material for: Transcriptome profiling of pumpkin (Cucurbita moschata Duch.) leaves infected with powdery mildew
Source: PLoS One. 2018 Jan 10;13(1):e0190175. doi: 10.1371/journal.pone.0190175 (PMC5761878; doi:10.1371/journal.pone.0190175)
Supplement: S1 Table — (DOC) [file pone.0190175.s005.doc]

**S1Table. The sequence of primers employed in this study**

| Gene | Gene ID | Forward (F) and Rorward (R) primer  5’→3’ |
| --- | --- | --- |
| MBF  MYB  bHLH87  BEE  bHLH61  HSF  ERF014  OFP  HSP70  glutaredoxin  EUL  MLO3  β-actin  SGT1  STDS  ABHD  WRKY21 | c101829_g1  c142659_g1  c66236_g1  c68108_g2  c71304_g1  c72139_g2  c146381_g1  c119105_g1  c115600_g1  c143537_g1  c44996_g1  c63983_g1  c8328_g1  c60148_g2  c71433_g2  c52777_g1 | F: TACCGACGGAGGTGAGGCAT  R: TGACGCCCAAGACCTTCTCCAT  F: CTCTCAGATGACCTCATTACCA  R: AGTCGGAGTTCTCGTAGTAACA  F:CAAACTCATATCAAACCAACAGA  R: TATCATCTCCTTCATTTGTGCTA  F: GTTGGGCTTTGGAAGGTTTG  R: GAGGAACAGCAGCTTGATTG  F: TCGGCGAAGTTTAATGTGGA  R: TGGACAGTGGACAGCAACAG  F: GTGAATTTGGACTCGAAGGTG  R: GTCCGATTTCAACGCCTCT  F: TGTCGTCCGATGGTGTCTA  R: GACTGAAGGGTATCAAGGTAAT  F: AAGAAAACCCAGCAGCCGA  R: ACCTGGTTGTTTCCGCCC  F: GAGGATATTGAGCGAATGTTG  R: CTTCAGCCCCAGAACCATAA  F: TGCCTTTGTTATGCAGTGGT  R: AGCGACATGACTTCGTTGGT  F: TTGGGTTTCGCCTCCGTCAT  R: CTTGGGAGTGGCTTGGATCA  F: TACTTTACCTCTTTATGCTCTGG  R: TCAATAATATCAAGATTGTTGAGC  F: TCTCTATGCCAGTGGTCGTA  R: CCTCAGGACAACGGAATC  F: ATTACCCAGAGCATTAGTGTCCC  R: TATCCTCGTCAGCGTCCTTGTAT  F: GCTTCACCAACGCTTTAGACATC  R: GCCATTAGAAGAAACGAGGAGTC  F: TCCTCCTGGTTCTATGGGTCTG  R:GACCGATCAGAGTCTCCTTGC  F: TCATAAACAAGCTGCTCC  R: CTCCCATCCATACTCAAC |
