# Supplementary material for: Transcriptome profiling of pumpkin (Cucurbita moschata Duch.) leaves infected with powdery mildew
Source: PLoS One. 2018 Jan 10;13(1):e0190175. doi: 10.1371/journal.pone.0190175 (PMC5761878; doi:10.1371/journal.pone.0190175)
Supplement: S2 Table — (DOC) [file pone.0190175.s006.doc]

**S2 Table. Output of the transcriptome sequencing for pumpkin**

| **Sample** | **Raw Reads** | **Clean reads** | **Clean bases** | **Error(%)** | **Q20(%)** | **Q30(%)** | **GC(%)** |
| --- | --- | --- | --- | --- | --- | --- | --- |
| PM_L241 | 72339512 | 70207406 | 10.53G | 0.02 | 95.38 | 89.25 | 46.48 |
| PM_L242 | 51821602 | 45744960 | 6.86G | 0.02 | 96.67 | 90.73 | 46.87 |
| PM_L481 | 73988674 | 70961286 | 10.64G | 0.02 | 97.01 | 92.93 | 46.14 |
| PM_L482 | 100847712 | 98582028 | 14.79G | 0.02 | 95.96 | 90.64 | 46.82 |
| W_L241 | 85820862 | 83885770 | 12.58G | 0.02 | 95.51 | 89.47 | 46.24 |
| W_L242 | 58942348 | 53892370 | 8.08G | 0.02 | 96.45 | 90.44 | 46.21 |
